# Supplementary material for: Co-infection mathematical model for HIV/AIDS and tuberculosis with optimal control in Ethiopia
Source: PLoS One. 2024 Dec 10;19(12):e0312539. doi: 10.1371/journal.pone.0312539 (PMC11630616; doi:10.1371/journal.pone.0312539)
Supplement: Attachment — Submitted filename: Authors response to Journal of PLOS ONE.pdf [file pone.0312539.s001.pdf]

Response to Reviewers

PONE-D-24-26378

Co-infection mathematical model for HIV/AIDS and tuberculosis with optimal control in Ethiopia

Dear Joshua Kiddy K. Asamoah, PhD,

Academic Editor,

PLOS ONE

Thank you for giving us the opportunity to submit a revised draft of the manuscript "Co-infection mathematical model for HIV/AIDS and tuberculosis with optimal control in Ethiopia" for publication in the Journal of PLOS ONE. We appreciated the time and effort that you and the reviewers dedicated to providing feedback on our manuscript. We gratefully thank you for the insightful comments on and valuable improvements to our paper. We have incorporated all of the suggestions made by the reviewers. We feel that in responding to these comments and suggestions, we have significantly improved the quality and readability of the paper. Those changes are highlighted within the manuscript. Please see below, in blue, for a point-by-point response to the reviewer's comments and concerns.

Note:

We have prepared the revised manuscript by using the PLOS ONE LaTeX template \documentclass[10pt,letterpaper]{article}, \usepackage[top=0.85in,left=1.5 in, right= 1.5cm, footskip=0.75in]{geometry}. Thus, the entire work in the manuscript seems condensed and the total page number is reduced.

Gneral Comments:

1. Is the manuscript technically sounds, and do the data support the conclusions?

   The manuscript must describe a technically sound piece of scientific research with data that supports the conclusions. Experiments must have been conducted rigorously, with appropriate controls, replication, and sample sizes. The conclusions must be drawn appropriately based on the data presented.

Reviewer #1: Partly

Reviewer #2: Yes

Authors' Response:

We believe that, the conclusion of the manuscript is drawn from both the analytic and numerical results of the model. The analysis of the model showed significant results. Thus, the manuscript has practically sound part of scientific research.

2. Has the statistical analysis been performed appropriately and rigorously?

Reviewer #1: N/A

Reviewer #2: N/A

3. Have the authors made all data underlying the findings in their manuscript fully available?

   The PLOS Data policy requires authors to make all data underlying the findings described in their manuscript fully available without restriction, with rare exception (please refer to the Data Availability Statement in the manuscript PDF file). The data should be provided as part of the manuscript or its supporting information, or deposited to a public repository. For example, in addition to summary statistics, the data points behind means, medians and variance measures should be available. If there are restrictions on publicly sharing data—e.g. participant privacy or use of data from a third party—those must be specified.

Reviewer #1: No

Reviewer #2: No

Authors' Response:

Thank you for pointing this out. We have provided all data's and supporting documents that are used for fining this scientific research. For instance, we have taken some data from:

- Ethiopia-national strategic plan tuberculosis and leprosy control (2013-2020/21): https://www.afro.who.int/publications/national-strategic-plan-tuberculosis-and-leprosy-control-2006-2013-ec-201314-2020
- WHO report, CDC, UNAIDS etc…

Hence, the data taken from those sources are used us to estimate the values of the parameters involved in the model.

In addition, we have taken direct data from Ministry of health in Ethiopia. We have uploaded it on public repository of the journal.

4. Is the manuscript presented in an intelligible fashion and written in Standard English?

   PLOS ONE does not copyedit accepted manuscripts, so the language in submitted articles must be clear, correct, and unambiguous. Any typographical or grammatical errors should be corrected at revision, so please note any specific errors here.

Reviewer #1: No

Reviewer #2: Yes

Authors' Response:

We corrected all English and typo mistakes of the manuscript.

Reviewer #1 Comments:

1. The abstract fails to justify the need for this research study. Also, the first statement in the Abstract is unnecessary. Remove it and reconstruct the abstract with necessary information. Be clear with the reason behind this work, how to go about it and the key results obtained.

Authors' Response:

Thank you so much reviewer #1 for your relevant comments.

We revised the abstract part of the manuscript. We addressed the points mentioned by the reviewer. Those changes are presented in the revised manuscript.

2. The novelty of this research is not clearly stated. Please do that.

Authors' Response:

We addressed the novelty, aim, purpose, and motivation of this research from paragraph 3-5 of page 4 in the revised manuscript.

3. The authors claim that "However, none of these authors have studied the extension of model [68] with HIV/AIDS cohorts and incorporating optimal control efforts". The referred article [68] was extended in 2022 Zhong-Kai et. al. by where the authors studied the HIV/AIDS-TB

co-infection model with optimal control by further considering age structure. Authors may find the article details here "Guo, Z. K., Huo, H. F., & Xiang, H. (2022). Optimal control of TB transmission based on an age structured HIV-TB co-infection model. Journal of the Franklin Institute, 359(9), 4116-4137".

Authors' Response:

We addressed the comment at paragraph 4 of page 5, but we can also clarify as:

Zhong-Kai et. al. didn't extend the model with 12 compartments like us. In addition, they didn't consider two latent cases such as high risk and low risk latent by treatment on their extended model. Further, they only considered two controlling strategies such as detection and treatment of latent cases and educational campaign to control the disease. Whereas, our model is totally differ from the model they developed and we considered four controlling measures such as preventive effort of TB, preventive effort of HIV, case finding for TB, and HIV treatment.

4. The authors said also that due to lack of data they picked some parameter values from literature. How true is this? Are the authors claiming that there is no data on HIV/AIDS or TB or their co-infection?

   Authors' Response:

Thank you for the comment. However, we have not said like that. We have taken some parameter values involved in the model from related literature as presented in table 2 of the manuscript. Some are directly taken from WHO annual report for a particular region Ethiopia. In addition, we have considered variables within a nation's population, such as the disease progress on age, gender, marital status etc… We also considered demographic models which are often used to detect and correct faulty data, impute missing data, and allow researchers to infer from partial data. Not surprisingly, this use of demographic models is applicable for a research in less developed countries, where quality data are scarce. However, over 90% of the values of the parameters are estimated based on the real data in Ethiopia.

5. In table 2, the authors have indicated that some parameters were fitted. Which year's population for Ethiopia was used for the estimation? Where from the value of N? What is the source of the data used for the estimation? Kindly consider coming out with an updated

parameter estimation section.

Authors' Response:

We said the values of most parameters are fitted based on the real data collected from Ethiopia. Look the number of confirmed cases in Ethiopia from 2021-2023 as shown in the Figure 3. The value of N is obtained from National TB and Leprosy strategic plan in Ethiopia which is cited in the reference section. That is $N = 102\ \ 468\,037$. Because N is very large, this can be formulated by $N = S + E + I + L + H + A + H_L + H_A + H_E + T + A_L + A_I$. The source of the data used for estimation are cited in the reference section and presented in Figure 3. We also attached the raw data collected file in supporting documents.

6. Authors should note that table labels should be placed on top of the table.

Authors' Response:

Thank you for the comment. We think the reviewer said captions not labels. We corrected and updated it.

7. The article has enormous grammatical errors. Please read through the entire work to correct them.

Authors' Response:

Thank you for your comment. We read all entire work and corrected all grammatical and typo errors.

8. All model assumptions should be supported with literature.

Authors' Response:

Thank you for your comment. We addressed the comments as shown at section 2 in the revised manuscript.

9. Though I see a section on positivity and boundedness. Unfortunately, I did not see any work on positivity. Please provide that.

Authors' Response:

Thank you for your comment. We addressed the comments as shown at section 3.1 in the revised manuscript.

10. Authors should be clear with the bifurcation analysis. Does the model undergo a forward or backward bifurcation? Where is the bifurcation graph?

Authors' Response:

Thank you for your comment. The backward bifurcation is emerged in the model. The analysis is presented at page 23 and the bifurcation plot is displayed at page 24 of the revised manuscript.

11. Under the control model, is early detection not more of a control mechanism than preventive?

Authors' Response:

The answer is **NO**. The optimal model is not described like that. Similar to educational campaign, early detection is one way of executing preventive mechanisms of TB or HIV/AIDS disease. Clearly, preventive measures are represented by $u_1(t)$ and $u_2(t)$ as:

- ✓ Preventive measures of TB disease ($u_1(t)$) take account of providing TB education program and early detection and isolation of TB infectious individuals.
- ✓ Preventive measures of HIV/AIDS disease ($u_2(t)$) take account of providing HIV/AIDS education program and early detection of HIV infected individuals.

Hence, early detection is one way of implementing preventive mechanisms but not considered as control variables like $u_1(t), u_2(t)\ldots$

Thus, we cannot compare early detection and preventive measure.

12. Conclusion of the work should be updated. The current form is too long and does not highlight the essential aspects of the work. What are the gaps in the work?

Authors' Response:

Thank you for your comment. We revised and updated the conclusion of the study. We also presented the essential aspect of the study. We addressed the gaps in the work both in the introduction part and conclusion section in the revised manuscript.

13. The introduction and model formulation sections require improvement. Please do that.

Authors' Response:

We have revised and enhanced the two sections. We also corrected English grammatical and typo mistakes. In addition, supportive references in the model assumption are cited.

Thank you so much the reviewer #1 again.

Reviewer #2 Comments:

Reviewer #2: I read the paper in details, the paper has a potential for publication provided the authors attends to the following suggestions:

1. The abstract should contain answers to the following questions: Why is this particular study important? What are the important results from this study different from others? What conclusions can be drawn from the results in this study?

Authors' Response:

Thank you so much reviewer #2 for your relevant comments and suggestion for our paper to publication in PLOS ONE. We addressed your comments as stated below.

We revised and updated the abstract of the study.

As stated in the abstract of the revised manuscript, this study has important public health lessons. The results found in the study will notify evidence based approaches to control the HIV-TB co-epidemic disease in Ethiopia. This is relevant for public and private health administrators. In addition, this will aids in the fight against HIV/AIDS, TB, and their co-infection policy-makers. Further, our study can impute for other countries under this disease burden to revise or update their disease control policy.

Our study is differing from other works published before. In this study, we developed a new co-infection mathematical model with 12 compartments by taking in to account TB cases: high risk exposed stage (E), low risk latent by treatment (L), high risk and low risk latent TB infected individuals co-infected with HIV. As far as our knowledge concerned, the two latent stages co-infected with HIV/AIDS were not considered in other co-infection models. Most models were developed considering with people are recovering from TB infection. However, we developed a TB-HIV co-infection model via assuming that TB infected individuals are not fully recovered. Which means tubercle bacilli cannot be 100% removed from TB infected individuals.

The proposed intervention strategies based on prevention of TB, prevention of HIV-AIDS, case finding for TB, and HIV treatment by ART are not analyzed in other previous co-infection models.

In addition, more parameter values are estimated based on the data collected in Ethiopia.

The abstract concluded by the main result of the study which is applying HIV/AIDS prevention efforts and TB case finding concurrently is the most cost-effective strategy to offer substantial relief from the burden of the pandemic in the community.

2. The literature review of epidemic models in this direction is too weak and must be improved meticulously, since there are many works in this direction.

   Authors' Response:

   Thank you for the comment. We have improved the literature review of the study as shown in the revised manuscript.

3. Support important model assumptions with biological evidences. Also state if the model is a new one or a modification of an existing model.

   Authors' Response:

   The model assumptions are updated with biological evidences. The model is a **new co-infection model** starting from **SEIL** tuberculosis model. The foundation is correctly cited.

4. What is the biological significance of the Basic reproduction number, as it regards to disease control? Also discuss Theorem 1 and why it important.

   Authors' Response:

   The basic reproduction number (R_0) is defined as the average number of secondary cases produced by a typical infectious in a completely susceptible population. Consequently, the value of R_0 allows to forecast if the disease will persist or vanish. If $R_0 < 1$, then the disease cannot spread in the community. Whereas, If $R_0 > 1$, then the disease spreads through the population. In our model, we found the values of R_0 using MATLAB which is $R_0 = \max\{R_T, R_H\}$, where $R_T = 2.3718$ and $R_H = 1.8907$. Thus $R_0 = 2.3718$. This shows the disease is endemic in the community. In addition, the phenomenon of backward bifurcation is occurred at $R_0 = 1$ as shown in Figure 2. Hence, the stable endemic equilibrium point co-exists with the stable disease free equilibrium point. This implies that the epidemiological condition of having $R_0 < 1$ is necessary but not sufficient condition to control HIV-TB co-infection in the populace. Thus, additional measures must be embraced to mitigate the burden of the disease.

   The necessary condition is to eliminate/minimize HIV-TB co-infection in Ethiopia, to reduce R_0, and to have $R_0 < 1$. The basis reproductive number, R_0, is a very useful quantity in mathematical analysis and epidemiological application.

The other comment is the important of Theorem 1 but now it is shifted to Theorem 2 in the revised manuscript. Similarly for Theorem 2 shifted to Theorem 3. Since, our co-infection model is a union HIV and TB sub-models. Hence, we have to cheek the validity of each disease model analytically.

Theorem 1: The DFE point of the HIV/AIDS sub model is locally asymptotically stable (LAS) if $R_H < 1$ and unstable if $R_H > 1$.

The theorem has epidemiological important, because if there is a small perturbation of the system (for example, a small number of HIV-infected individuals is introduced into the population), then, after some time, all state solutions in the system will return to the Disease Free Equilibrium (DFE) point. Thus, **Theorem 1** helps us to comprehend that the number of the infected population will gradually become lower and lower if the new infection created by an individual during his/her infection period is less than one.

Whereas, **Theorem 3**: The globally stability of Endemic Equilibrium (EE) of the model for $R_H > 1$. However, a big perturbation may be able to drive the system to a different behavior e.g. convergence to the EE point. If the stability of the EE point is global, no matter the size of the perturbation, persistence of the disease becoming endemic in the population. Thus, the epidemiological interpretation of Theorem 3 is that, there is at least a certain number of infected population if the new infection created by an individual during his/her infection period is more than one.

5. What is the epidemiological significance of theorems 1, 2? Be more detailed in discussing the many mathematical analyses. I advise the authors to check some recent papers with useful approach for analysis.

    Authors' Response:

    Thank you for your question and recommendations. We have addressed the Theorem 1 and 2 in answer question 4.

    The articles which are recommended by reviewer 2 are listed. We have read and cited them in the revised manuscript.

Thank you so much the reviewer #2 again.

Finally, we look forward to hearing from you in due time regarding our revised submission and to respond to any further questions and comments you may have.

Sincerely,

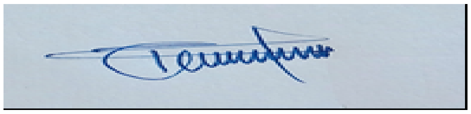

Tigabu Kasie Ayele

Corresponding Author

Date September 25/ 2024
